# Sexual Behaviour and Human Papillomavirus-Positive Oral Cavity and Oropharyngeal Cancer: An Irish Perspective

**DOI:** 10.7759/cureus.11410

**Published:** 2020-11-10

**Authors:** Thomas J Crotty, Emma Keane, Grainne Cousins, Sinead Brennan, John Kinsella, Tom Moran

**Affiliations:** 1 Department of Otolaryngology, Head and Neck Surgery, Mater Misericordiae University Hospital, Dublin, IRL; 2 Department of Pharmacy, Royal College of Surgeons in Ireland, Dublin, IRL; 3 Department of Radiation Oncology, St. James University Hospital, Dublin, IRL; 4 Department of Otolaryngology, Head and Neck Surgery, St. James University Hospital, Dublin, IRL

**Keywords:** head and neck cancer, human papillomavirus, sexual behaviour, oropharyngeal scc

## Abstract

Background

Characterization of the sexual behaviours and lifestyle factors associated with human papillomavirus (HPV)-positive oral cavity and oropharyngeal squamous cell carcinoma (OPSCC) is crucial to optimal counselling. Our study aims to investigate the relationship between sexual behaviours, lifestyle factors and HPV-positive OPSCC in an Irish population.

Methods

We performed a case-control study of 60 patients with newly diagnosed HPV-positive and HPV-negative oral cavity and OPSCC.

Results

Oral sexual activity was more common in the HPV-positive tumour subgroup; however, this association was insignificant on multivariate analysis. No association between age of onset of sexual activity, number of sexual partners or practicing anal sex and HPV-positivity was found. The HPV-positive tumour subgroup had significantly less tobacco use than their HPV-negative counterparts (OR 0.93, 95% CI 0.90-0.97).

Conclusion

The emergence of HPV-positive OPSCC means head and neck surgeons must adopt new roles as counsellors of sexually transmitted disease, in addition to their previous role of delivering a cancer diagnosis.

## Introduction

Head and neck cancer is responsible for 3% of all invasive cancers diagnosed in Ireland each year [1]. Patients frequently present with locally advanced disease, resulting in approximately 213 deaths annually [1]. Traditionally, the majority of these malignancies are caused by the multiplicative effects of excessive alcohol consumption and tobacco use. Recent public health strategies targeting tobacco control has led to a decrease in the incidence of head and neck cancer globally [2]. An exception to this is oropharyngeal squamous cell carcinoma (OPSCC), a subgroup of head and neck cancer, which has rapidly increased in incidence over the past four decades [3]. Human papillomavirus (HPV) 16, a sexually-transmitted oncogenic strain of HPV and well-established cause of anogenital cancer, is recognised as the main etiological factor driving this trend. Although no detailed figures exist in Ireland, figures from the United Kingdom estimates that approximately 50%-55% of OPSCC’s diagnosed each year are caused by HPV, surpassing alcohol and tobacco as the most common cause for oropharyngeal cancer [4].

OPSCC is broadly divided into two categories based on p16 expression, as represented by the 8th edition of the American Joint Committee on Cancer (AJCC) staging system: HPV-positive (p16 overexpression) and HPV-negative OPSCC [5]. HPV-positive tumours possess highly carcinogenic HPV-16 DNA integrated in tumour cell nuclei expressing oncoproteins E6 and E7. These viral oncoproteins degrade tumour suppressor genes p53 and Rb respectively, leading to loss of cell cycle control. In contrast, HPV-negative OPSCC is caused by a wide variety of p53 and Rb mutations [6]. Patients diagnosed with HPV-positive tumours are usually younger, have minimal exposure to tobacco or alcohol and are more likely to have a history of multiple sexual encounters [7]. Clinically, HPV-positive OPSCC typically presents as a localized tonsillar or a base of tongue lesion with early lymph node involvement [8]. In contrast, HPV-negative tumours present as a locally advanced primary tumour without spread to regional lymph nodes [8]. Despite the frequent involvement of regional lymph nodes, HPV-positive tumours have a better prognosis due to their responsiveness to cisplatin-based chemotherapy with concurrent radiotherapy [7].

The recent upsurge in the incidence of OPSCC is speculated to be a consequence of the maturation of the “baby boom” generation, leading to a sexual revolution [9]. Since the 1970’s there has been a documented change in sexual norms with a decrease in the age of first sexual intercourse and a concurrent rise in the lifetime number of sexual partners [10]. It is believed that the changing trends in sexual activity have facilitated the transmission of high-risk HPV into the oral cavity and oropharynx, where it preferentially settles in the reticulated crypts of the palatine and lingual tonsils [11]. Case-control studies have highlighted certain sexual factors, such as increased number of lifetime vaginal or oral sexual partners, younger age at first sexual intercourse, and history of unprotected sex as having a positive association with persistent oral HPV-16 infection, HPV-16 positive serology and subsequent development of HPV-positive OPSCC [12]. Of which, the most strongly and consistently associated risk factor is the number of oral sexual partners. However, other demographic and lifestyle factors including male gender and smoking status, also have a strong effect on the risk of developing OPSCC [13].

The recognition of HPV-positive OPSCC as a sexually transmitted disease has led to a major shift in the traditional management of patients with head and neck cancer. The typical patient has evolved. Previously, patients were 60-year-old males with chronic exposure to alcohol or tobacco. Currently, patients are 50-year-old males without a significant smoking or alcohol history, and without other co-morbidities. This transformation has many implications for the diagnosing and treating clinician. Initial counselling now plays a pivotal role in a patient’s understanding of both their cancer diagnosis and its association with HPV. Previous studies have shown a diagnosis of HPV-positive OPSCC may lead to significant psychosocial issues concerning fidelity, and concern regarding the transmissibility of the virus to a patient’s partner [14]. In addition, patients with HPV-positive OPSCC have a better prognosis, allowing them to concentrate on the psychosocial consequences of the diagnosis.

Despite the recent surge of international data associating sexual behaviours, HPV-16 and OPSCC, a significant knowledge gap still exists in relation to the sexual behaviours of patients with OPSCC in an Irish population. Due to the increasing incidence of HPV-positive OPSCC, there is a growing importance in recognising this new demographic profile and characterizing the behaviours that put these patients at risk for developing the disease, so that we can counsel patients more effectively. In this study, we endeavor to build on the body of knowledge by comparing the sexual behaviours of patients diagnosed with squamous cell carcinomas of the oral cavity and the oropharynx in an Irish hospital setting.

## Materials and methods

This retrospective case-control study was performed in St. James’s Hospital, Dublin, over a one-year period. Eligible cases included patients diagnosed with oral cavity and oropharyngeal squamous cell carcinomas. Histology was confirmed by histological analysis of formalin-fixed, paraffin-embedded biopsy specimens taken as a part of their standard treatment. The case group was identified using the p16 (p16INK4a) biomarker on immunohistochemistry as a surrogate for HPV-positivity, while the control group consisted of patients with p16 negativity. 30 patients were identified as eligible case subjects. Control group consisted of 30 unmatched patients with p16 negativity on immunohistochemistry. 

The study protocol was approved by St. James Hospital institutional review board and a written informed consent was obtained from all patients. All participants completed a structured interview at the Ear, Nose and Throat outpatients department during routine follow-up by the same interviewer. Reporting was in accordance with the recommended principles of the Strengthening the Reporting of Observational Studies in Epidemiology (STROBE) statement for case-control studies. Data were elicited on demographic characteristics, lifestyle factors (tobacco use and alcohol consumption), and sexual behaviours. Data regarding sexual behaviours included sexual orientation, age at first sexual intercourse, number of lifetime sexual partners, having ever participated in vaginal, oral-genital, oral-anal or anal sex, clinical history of sexually transmitted disease, regular correct condom use, and if their partner had a previous diagnosis of cervical dysplasia. Tobacco use data was measured by the mean number of cigarettes in pack years. Data on alcohol consumption was reported as the mean number of alcohol units consumed per week. 

Statistical analyses were performed using Stata software, version 12.1 (StataCorp, College Station, TX). Case-control comparisons were made between the two groups. A series of univariate and multivariate analyses were performed. Multivariate analyses involved hierarchical logistic regression to identify associated factors. The multivariate models were used to calculate adjusted odds ratios, their 95% confidence intervals, and the corresponding p-values on covariates that revealed a statistically significant univariate p-value (p < 0.05).

## Results

HPV-positive tumours were most commonly detected at the tonsils and the tongue base, accounting for 77% of the HPV-positive tumours. The most common sites in the HPV-negative group were the anterior tongue and tongue base, although location of HPV-negative tumours were more widely distributed compared to HPV-positive tumours. The tumour distribution of our cases and controls is shown in Table 1.

 

**Table 1:** Oral cavity and oropharyngeal cancer by location HPV, human papillomavirus

Tumour Location	HPV-Positive, N = 30 (%)	HPV-Negative, N = 30 (%)
Tonsil	14 (47%)	2 (7%)
Gingiva	2 (7%)	1 (3%)
Posterior Pharyngeal Wall	4 (13%)	1 (3%)
Base of Tongue	9 (30%)	7 (23%)
Anterior Tongue	0	10 (33%)
Retromolar Trigone	1 (3%)	2 (7%)
Buccal Mucosa	0	2 (7%)
Palate	0	3 (10%)
Alveolus	0	2 (7%)

The mean age of the participants interviewed was 59.5 years. The HPV-positive group was slightly younger overall, but this was not significant. The majority of the study population was male (76%), with marginally more males in the HPV-positive group than the HPV-negative group (40% v 36.7%). 78% of patients, from both groups, were in a relationship at the time of this study. 

Expectedly, a positive association was seen between tobacco use and the HPV-negative subgroup, with the mean smoking pack-year in this group being 56 pack-years (OR 0.93; 95% CI 0.90-0.97). In contrast, the mean smoking pack-years for the HPV-positive group was 12.7 pack-years, including 15 participants who have never smoked. This association remained statistically significant after adjusting for confounding variables (OR 0.93; 95% CI 0.89 - 0.97; p < 0.01). The mean alcohol consumption per week was significantly higher in the HPV-negative cohort overall (OR 0.98, 95% CI, 0.96-1.00), but this did remain independently significant on multivariate regression analysis (OR 1.00; 95% CI 0.98-1.01). 

In terms of sexual behaviour, there were no significant differences between age of onset of sexual activity (OR 0.98; 95% CI 0.85-1.13) or whether the participant was sexually active or not at the time of diagnosis (OR 2.62; 95% CI 0.92-7.46). Lifetime number of sexual partners was 17.5 in the HPV-positive cohort compared with 4.7 in the HPV-negative group. There was a significant difference in multivariate analysis (OR 1.23; 95% CI 1.02-1.49); however, this was not significant on univariate analysis (OR 1.12; 95% CI 1.00-1.25), which may be the result of a dependent variable causing a suppression effect. All participants had performed vaginal intercourse and 55% reported being sexually active at the time of diagnosis.

Table 2 demonstrates the significantly positive association between oral sexual practices and patients who have developed HPV-positive OPSCC (OR 5.23, 95% CI, 1.66-16.51). 80% of the HPV-positive group had ever practiced oral sex compared with 43% of the HPV-negative group. However, after controlling for potential confounders, we observed an elevated, but insignificant association (OR 3.10; 95% CI, 0.54-17.66). Nearly 20% of all patients had practiced anal sex, but no difference between the two groups was demonstrated in this study (OR 0.80, 95% CI, 0.22-2.97). Although twice the number of subjects participated in oral-anal sex in the HPV-positive group, the sample size was too small to (N = 4) to establish significance (OR 2.15; 95% CI 0.36-12.76). No significant association was seen between the groups with regards to rates of sexually transmitted disease, correct use of condoms or exposure to a partner with previous diagnosis of cervical dysplasia or malignancy.

**Table 2:** Associations of oral cavity and oropharyngeal cancer with demographic factors, lifestyle factors and sexual behaviours *Adjusted odds ratio from multivariate analysis. OR, odds ratio; CI, confidence interval; STD, sexually transmitted disease.

Dependent Variable	HPV-Positive, N = 30	HPV-Negative, N = 30	Overall N = 60	Unadjusted OR (95% CI)
Age	58.9 yr	60.1 yr	59.5 yr	0.99 (0.94 – 1.04)
Gender				
Male	24 (40%)	22 (36.7%)	46 (76.7%)	1.00
Female	6 (10%)	8 (13.3%)	14 (23.3%)	0.96 (0.21 – 2.30)
Alcohol and Tobacco Use				
Tobacco use (pack-years)	12.7	56.1	34.4	0.93 (0.90 – 0.97) 0.93 (0.89 – 0.97)*
Never tobacco use	15 (50%)	1 (3.3%)	16 (26.6%)	-
Alcohol (units per week)	22.0	69.5	45.75	0.98 (0.96 – 0.99) 1.00 (0.98 – 1.01)*
Relationship Status				
With partner	25 (83.3%)	22 (73.3%)	47 (78.3%)	1.00
Not with partner	5 (16%)	8 (26%)	13 (21.6%)	1.41 (0.45 – 4.45)
Sexual Behaviour				
Onset of sexual activity	18.6 yr	18.9 yr	18.8 yr	0.98 (0.85 – 1.13)
Lifetime number sex partners	17.5	4.7	11.1	1.12 (1.00 – 1.25) 1.23 (1.02 – 1.49)*
Sexual partners in 5 yr before diagnosis	1.1	1	1.04	1.08 (0.68 – 1.73)
Sexually active at time of diagnosis	20 (66.7%)	13 (43.3%)	33 (55%)	2.62 (0.92 – 7.46)
Ever vaginal sex	30 (100%)	30 (100%)	60 (100%)	1.00
Ever anal sex	5 (16.6%)	6 (20%)	11 (18.3%)	0.8 (0.22 – 2.97)
Ever oral-genital sex	24 (80%)	13 (43.3%)	37 (61.6%)	5.23 (1.66 – 16.51) 3.07 (0.65 – 14.50)*
Ever oral-anal sex	4 (13.3%)	2 (6.6%)	6 (10%)	2.15 (0.36 – 12.76)
Regular use of condoms correctly	4 (13.3%)	5 (16.6%)	9 (15%)	0.77 (0.19 – 3.20)
Previous diagnosis STD	6 (20%)	4 (13%)	10 (16.6%)	1.63 (0.41 – 6.47)

## Discussion

This is the first study investigating the differences in sexual behaviours in patients diagnosed with oral cavity and OPSCC in a representative sample of the Irish population. Our data elucidated a trend towards significance in patients engaging in oral sexual practices and the development of oral cavity and OPSCC, as seen in previous case-control studies [12]. In addition, evidence of an alternative pathway for the development of OPSCC was demonstrated via individuals with HPV-positive tumours, who had a reduced exposure to tobacco, a well-established risk factor for head and neck cancers. Despite a small sample size, our results, in the context of previous similar case-control studies, highlights the changing landscape of head and neck oncology and the role physicians must adopt to counsel patients on their diagnosis of cancer, as a result of a sexually transmitted disease. 

As expected, the majority of HPV-positive OPSCC were located in the tonsils and tongue base, which is likely due to the crypt epithelium present in lymphoid tissue which provides an entry point for HPV [11]. Another potential contributor to the increased incidence of HPV-mediated tonsillar cancer is the decline in tonsillectomy rates over the past 50 years [15]. A recent study found that patients with a HPV-positive OPSCC at a non-tonsil site were much more likely to have had a previous tonsillectomy, suggesting if tonsils are present, they are a risk factor for developing primary tonsillar OPSCC [15]. In both groups, oral cavity and OPSCC were more common in males. The ratio of male:female was further magnified when looking at HPV-positive tumours with a magnitude of 4:1 (M:F). This can be, at least partially, explained by the higher number of lifetime sexual partners in men, however, even when this variable is matched men seem to be more at risk [16]. The leading theory postulates that women may mount a more robust immune response to oral HPV infection [17]. Unlike previous studies, the HPV-positive group was not found to be significantly younger than their HPV-negative counterparts, despite changes in sexual behaviours amongst recent birth cohorts being one of the key hypotheses for the younger age at diagnosis of HPV-positive OPSCC patients [16]. Importantly, one of the key supportive arguments for the utilization of reduced-dose radiotherapy in HPV-positive OPSCC patients is to avoid the long-term toxicities in younger, medically fit patients [18]. The lack of a significant age discrepancy between the two subgroups may be an example of the increasing prevalence of HPV-positive OPSCC’s amongst older cohorts also [19].

In our cohort, we found those who had engaged in oral sex were nearly twice as likely to have an HPV-related cancer (80% vs 43.3%). Despite this seemingly strong relationship, when factors such as tobacco use, alcohol consumption, age, and gender were controlled, having practiced oral sex failed to remain independently significant (OR 3.10; 95% CI: 0.54-17.66). It is plausible that the current study was underpowered to detect a positive association when adjusting for confounders. Other case-control studies demonstrated an exposure-response relationship between number of oral sexual partners and HPV-related OPSCC [12]. In addition, the HPV-positive group in this study did not detect a significantly higher number of lifetime sexual partners or younger age at first sexual intercourse. In comparison, a landmark study by D’Souza et al. [12] found that sexual intercourse before the age of 18, and a higher number of lifetime sexual partners increased the risk of being diagnosed with HPV related cancer. One of the main characteristics that define the HPV-positive group in the literature is reduced exposure to tobacco and alcohol [20]. In this study, the number of tobacco pack-years in the HPV-negative group was 440% greater than the HPV positive group (56.1 vs 12.7). Moreover, 50% of the HPV-positive group were non-smokers compared with only 3.3% of the HPV-negative group, a higher figure than documented in other studies [20]. The remaining 50% of HPV-positive patients, had an average of a 25 pack-year smoking history. This highlights the finding that a half to two-thirds of HPV-positive patients can be current or ex-smokers at diagnosis [21]. Although the HPV-driven subtype conveys a more favourable prognosis, continuing tobacco use significantly increases the risk of disease progression [22], as well as having a negative effect on flap complications [23]. Therefore, it is of paramount importance when counselling patients with suspected or confirmed HPV-related cancer, that smoking cessation confers a significant survival advantage. The HPV-positive group consumed an average of 22.03 units of alcohol per week, narrowly above the recommended maximum number of units for men, compared to 69.53 units per week for the HPV-negative cohort. Although trending towards significance, this was not significant when controlling for confounding factors.

The primary limitation of this study was a lack of power reducing the chance of detecting a significant result. Larger studies, such as the International Head and Neck Cancer Epidemiology (IHANCE) consortium, undertook a pooled analysis, including 5642 head and neck cancer cases and 6069 controls from several different countries [24]. Positive associations between HPV-positive OPSCC and numerous patterns of sexual behaviour were found, unlike our study which had a relatively small sample in comparison. Other limitations include the relatively heterogenous cohort. It would be advantageous for future studies to focus on a specific subset of upper aerodigestive cancer, with matched controls. 

The aim of this study was to further characterize the sexual behaviours of patients with HPV-positive oral cavity and OPSCC in an Irish population, so that we could counsel them more effectively regarding their diagnosis. Despite a plethora of research internationally providing evidence of this association [12,16,24], no research on this topic has yet been conducted on an Irish population. This is of real and practical value to counselling a patient regarding a diagnosis of HPV-positive OPSCC. When a clinician delivers a cancer diagnosis, it creates distress and anxiety for the patient, which may then be amplified by guilt and self-blame when the malignancy is linked to a sexually transmitted disease [25]. Reich et al. emphasized the importance of minimizing self-blame by ensuring the patient understands that HPV-16 can be shared amongst long-term partners, without the occurrence of infidelity, despite its link to increased lifetime of sexual partners and unprotected sexual intercourse [26]. It is common for patients to have concerns regarding the likelihood of their partner developing an HPV-related cancer. Although studies have shown a small increased risk of developing upper aerodigestive cancer from a female partner with cervical cancer [14], the reliable trend of partners developing an HPV-related malignancy from HPV-OPSCC has not been shown [27]. It is equally important to note that although HPV-driven cancer improves your prognosis, continuation of smoking and/or alcohol consumption may interact synergistically to worsen survival outcomes and must continue to be addressed during consultation [14].

## Conclusions

The development of an association between HPV and oral cavity and OPSCC has undoubtedly changed the landscape of head and neck oncology. Characterizing risk factors is an integral part of furthering our knowledge and obtaining the necessary information to adapt to this change. Head and neck surgeons must adopt a patient-centered role in counselling patients regarding sexually transmitted diseases, as well as the traditional role of diagnosing and treating cancer.
